# Rapid resolution of severe exudation in uveal effusion syndrome with anti-vascular endothelial growth factor alone in a case of bilateral nanophthalmos: a case report

**DOI:** 10.1186/s13256-021-03101-z

**Published:** 2021-10-19

**Authors:** Li Song, Fangtian Dong, Changxian Yi

**Affiliations:** 1 ChaoJiu Ankang Ophthalmic Hospital, DaTong, 037006 Shan Xi Province People’s Republic of China; 2 Tongren Hospital, Beijing, People’s Republic of China; 3 Chao Jiu Ophthalmic Hospital, Chifeng, 024000 Inner Mongolia People’s Republic of China; 4 Zhongshan Ophthalmic Center, University of Sun Yet-san, Xian Lie Nan Lu, 54, Guangdong Guangzhou, People’s Republic of China

**Keywords:** Case report, Nanophthalmos, Uveal effusion syndrome, Treatment of UES, Anti-VEGF

## Abstract

**Background:**

Uveal effusion syndrome is a rare disease characterized by exudative detachments of the choroid, ciliary body, and retina. Various surgical procedures and nonsurgical strategies have been described to treat uveal effusion syndrome with limited success. The treatment for uveal effusion syndrome remains a serious challenge for clinicians. To the best of our knowledge, no previous report has described a severe uveal effusion syndrome patient with nanophthalmos treated by using an anti-vascular endothelial growth factor agent alone. We report here one such case with unexpected positive results.

**Case presentation:**

A 30-year-old Chinese male patient presented with painless vision loss in both eyes that had persisted for 2 months. Examination of the right eye revealed a best corrected visual acuity of 0.03; the best corrected visual acuity of the left eye was finger count/20 cm. The intraocular pressure was normal on both eyes. A-scan revealed an right eye axial length of 15.88 mm and a left eye axial length of 16.21 mm. In the right eye, half of the peripheral choroid and nearly three-fourths of the retina were detached. The left fundus was not visible because of the total retinal detachment located just behind the lens, which could be clearly observed directly with a slit lamp. Considering all the possibilities and available treatments as well as the patient’s intentions after discussion, we first administered an intravitreal injection of ranibizumab 0.5 ml into both eyes. The patient’s visual perception improved 3 days after the injection. One month later, most of the effusion under the choroid and retina was absorbed. Visual acuity improved from finger count to 0.05 in both eyes, and vision quality was remarkably improved. Encouraged by this good result, the patient opted to undergo a second injection 1 month later. Choroidal and retinal detachment completely vanished 30 days after the second injection.

**Conclusions:**

Using an anti-vascular endothelial growth factor agent alone may be a potentially effective and safe method for managing some types of uveal effusion syndrome, such as in nanophthalmos. The injection may be administered before considering more aggressive procedures in some uveal effusion syndrome patients.

## Background

Uveal effusion syndrome (UES) is a rare, severe ocular condition that may threaten the sight and is characterized by exudative detachment of the choroid, ciliary body, and retina, thought to result from a congenital anomaly of the sclera involving increased scleral thickness and disorganization of scleral collagen fibers [1–3], which impairs posterior segment drainage by compressing the vortex vein and reducing the permeability of the sclera to macromolecular substances [1, 4, 5]. There are several other theories regarding the pathogenesis of UES. Jackson *et al.* proposed that the formation of effusion in the suprachoroidal space is osmotic fluid retention, in which a reduction in protein escape from the choriocapillaris is a key factor [6]. This potential problem is circumvented in healthy eyes because proteins can diffuse across the sclera [7]. A recent study also revealed that there is an increase in IL-6 and IL-8 VEGF in the aqueous humor, which supports the hypothesis that extra-effusion from abnormal permeability of choroid capillaries and inflammatory elements maybe involved in UES [8–10]. Based on the mechanism proposed, a number of surgical procedures, in particular full or partial thickness multisclerectomy, have been promoted [11–14]. However, some patients have been successfully treated with systemic/topical steroids [15], NSAIDs [16], latanoprost, and oral acetazolamide topical bromfenac [17, 18]. Anti-VEGF agents combined with surgical intervention have been reported as a treatment option [9, 10]. However, the exact mechanism underlying UES and rational treatment strategies have yet to be clarified. Each of the treatments available has limits and potential side effects [19]. UES is still widely considered a major challenge to clinicians.

To the best of our knowledge, no previous report has been published regarding the treatment of UES by using an anti-VEGF agent alone with quick improvement observed. We report here the case of one patient with bilateral nanophthalmos with severe retinal and choroidal detachment in both eyes. He was treated with an intravitreal injection of the anti-VEGF agent ranibizumab 0.05 ml alone, and a rapid resolution of effusion was observed in both eyes.

## Case presentation

A 30-year-old Chinese male patient presented with acute blurred vision in both eyes that had persisted for 2 months. The patient’s condition was painless with no photophobia, headache, or other irritating ocular symptoms. The patient had poor vision at birth; however, he was capable of walking and managing everyday life independently. He had minimal visual acuity, and his exact visual acuity before the onset of UES was unknown as the patient had no reliable record of his vision despite knowledge that his eyes were abnormal. The patient attended primary school for only 3 years before his vision loss prevented him from continuing schooling. He estimated that his best lifelong VA was 0.05–0.1, and this value was assumed to represent his normal vision before the occurrence of UES. The patient was admitted to the hospital with a diagnosis of retinal detachment in both eyes, and his treatment was provided at minimal cost due to the new antipoverty funding movement in China. There were no other obvious medical conditions or finding relevant to the patient’s ocular condition. There was no family history of any similar eye disease.

### Ocular examination

**Fig. 1:**
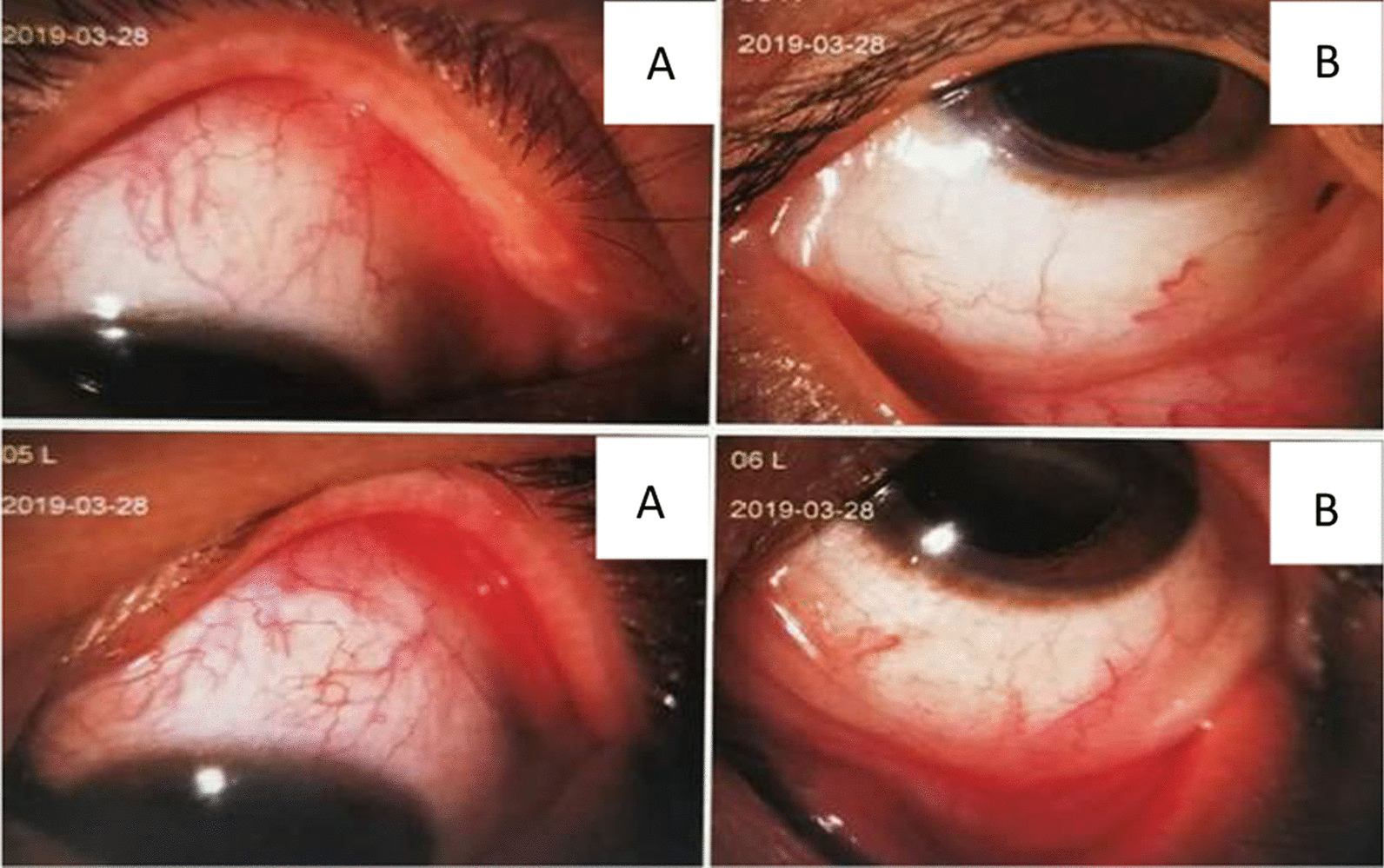
Exterior part of both eyes under slit lamp before treatment (upper **A** and **B** represent right eye; the lower ones represent left eye). Deep vessels of conjunctiva were slightly engorged and distorted. Cornea was transparent

**Fig. 2:**
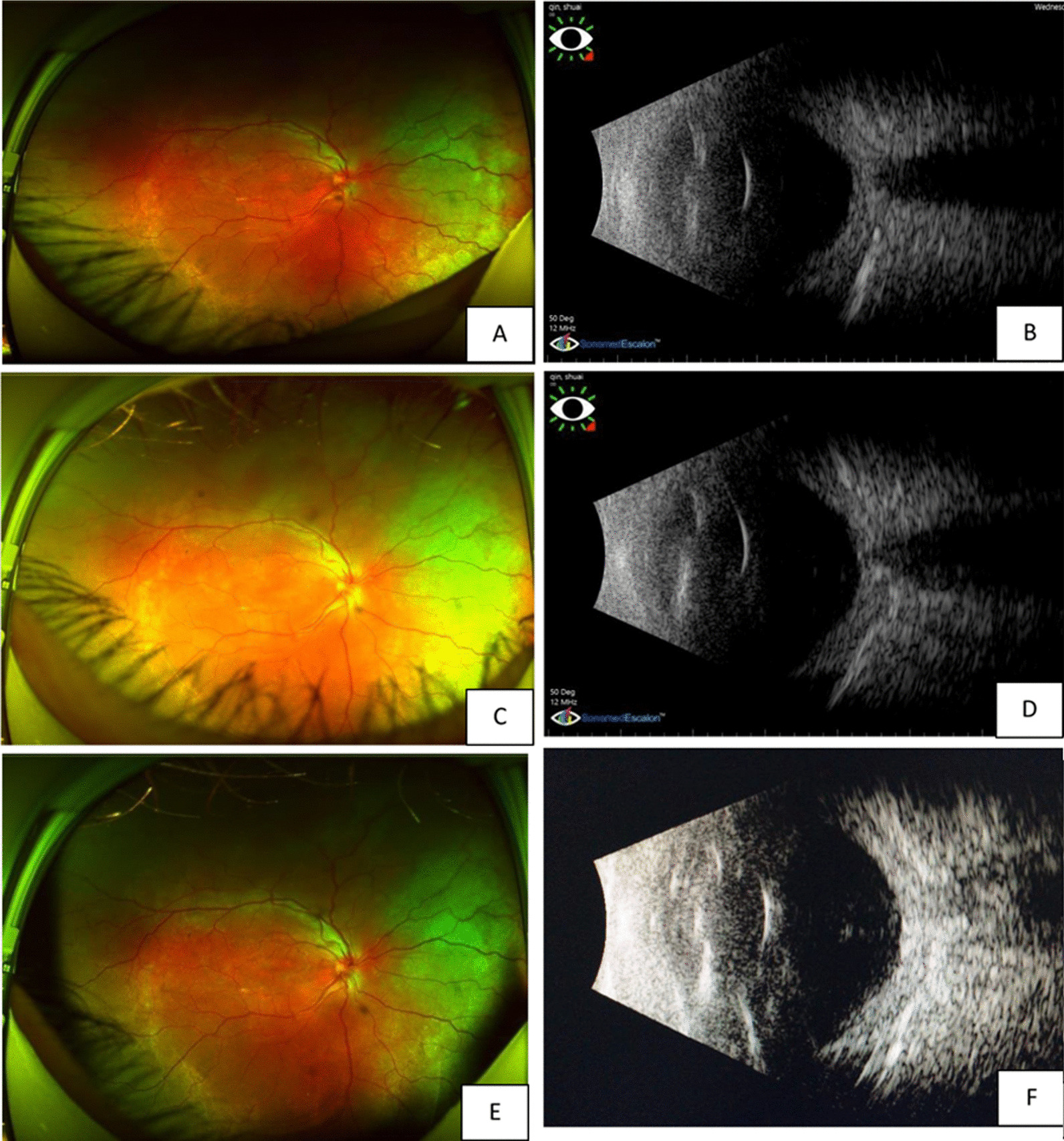
The fundus photos taken by Optos scanning laser fundus camera and B-scan of the right eye at different time points. **A** and **B** are color photo and B-scan before anti-VEGF; **C** and **D** are fundus and B-scan at 1 month after treatment; **E** and **F** are 2 months post-treatment

**Fig. 3:**
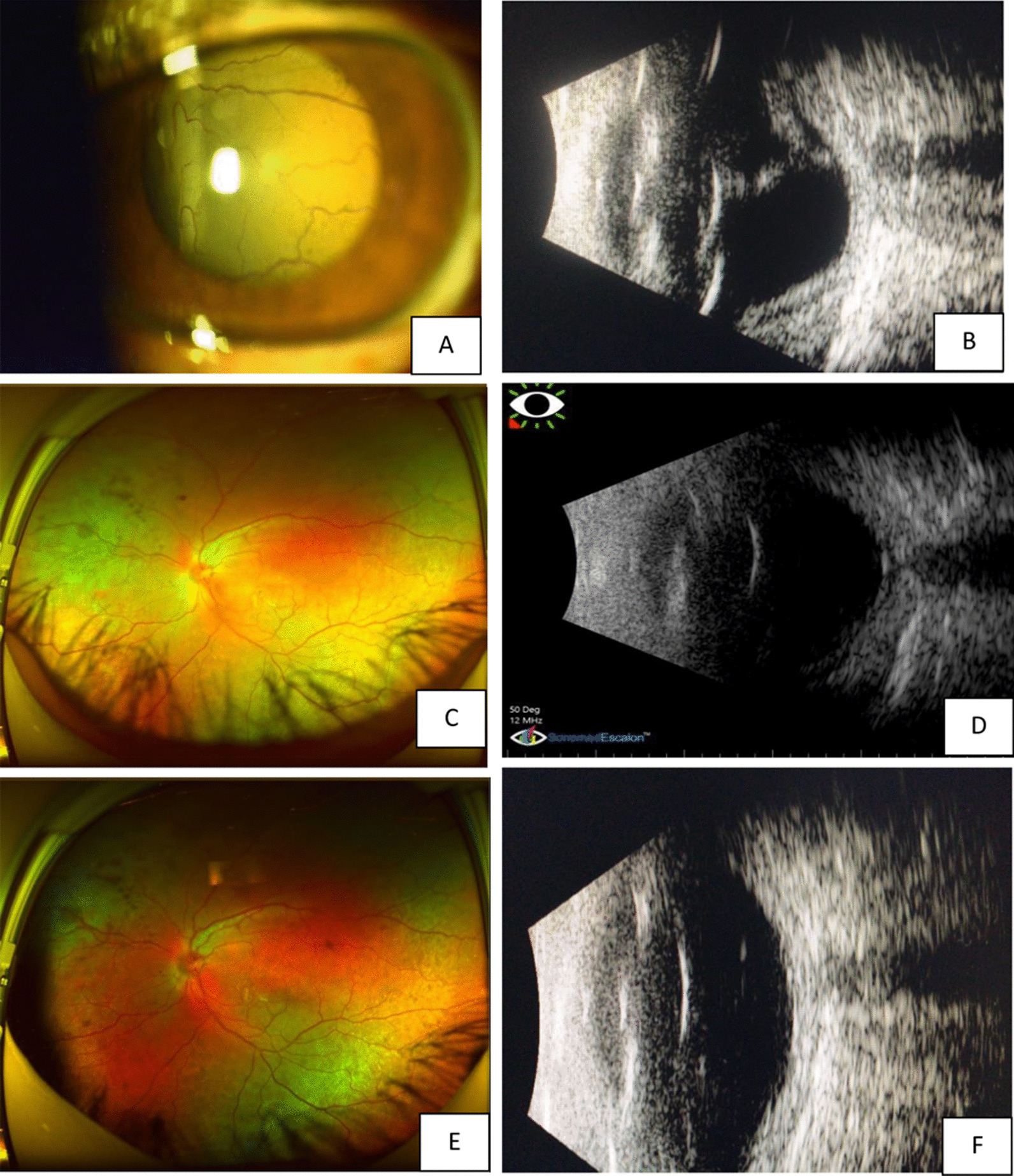
The fundus photos taken by Optos scanning laser fundus camera and B-scan of the left eye at different time points. **A** and **B** are color photo and B-scan before anti-VEGF; **C** and **D** are fundus and B-scan at 1 month after treatment; **E** and **F** are 2 months post-treatment

Examination of the right eye (OD) revealed a VA of FC/20 cm with a best corrected visual acuity (BCVA) of 0.03 (+16.00DS/+1.25DCX75 = 0.03); the VA of the left eye was FC/20 cm and was not improved by correction. The intraocular pressure (IOP) was 15 mmHg (OD) and 17 mmHg (OS). A-scan revealed an OD eye axial length of 15.88 mm and an OS eye axial length of 16.21 mm. No conjunctival hyperemia was observed, but the deep conjunctival vessels were engorged and distorted, and the cornea was transparent (Fig. 1). The patient was negative for keratic precipitates and flares, and no cells were observed in the anterior chamber (AC). The depth of the central anterior chamber was 2.9–3.0 mm in both eyes as measured by A-scan. The pupils were round and 3 mm in diameter. Direct and indirect pupil reactions existed but were remarkably reduced; lenses were clear in both eyes. In the right eye, the vitreous cavity was clear, there was no visible inflammation, and the optic disc was slightly dysplastic and crowded with normal color. The ratio of cup/disc is 0.3 and artery/vein is 2/3, and the foveal reflection disappeared with a mild fold appearance at the macula. Partial peripheral choroidal detachment was present; the middle and far peripheral retina was detached (Fig. 2). The left fundus was not visible because of the total retinal detachment located just behind the lens that could be clearly observed directly using a slit lamp (Fig. 3).

There was also no obvious sign of uveitis or scleritis in either eye. The intraocular pressure was normal even with severe detachment of the retina and choroid. Considering the patient’s extremely short eye axial length and after excluding the other possible conditions, the diagnosis of UES associated with congenital nanophthalmos was established.

### Treatment

Taking into consideration that nanophthalmos was present in both eyes and that the patient had had very poor vision since childhood, an aggressive surgical intervention was not considered to be capable of significantly improving visual acuity. Additionally, the patient was unwilling to risk further vision loss as, with his minimum VA, he was able to lead a relatively independent life. We decided intravitreal injection of the anti-VEGF agent ranibizumab 0.5 ml in both eyes instead of performing multisclerectomy, which is a more widely applied technique but represented a higher risk of complications in this case. Other auxiliary medicines administered for UES include vitamin tablets and steroid eye drops.

## Results

**Fig. 4:**
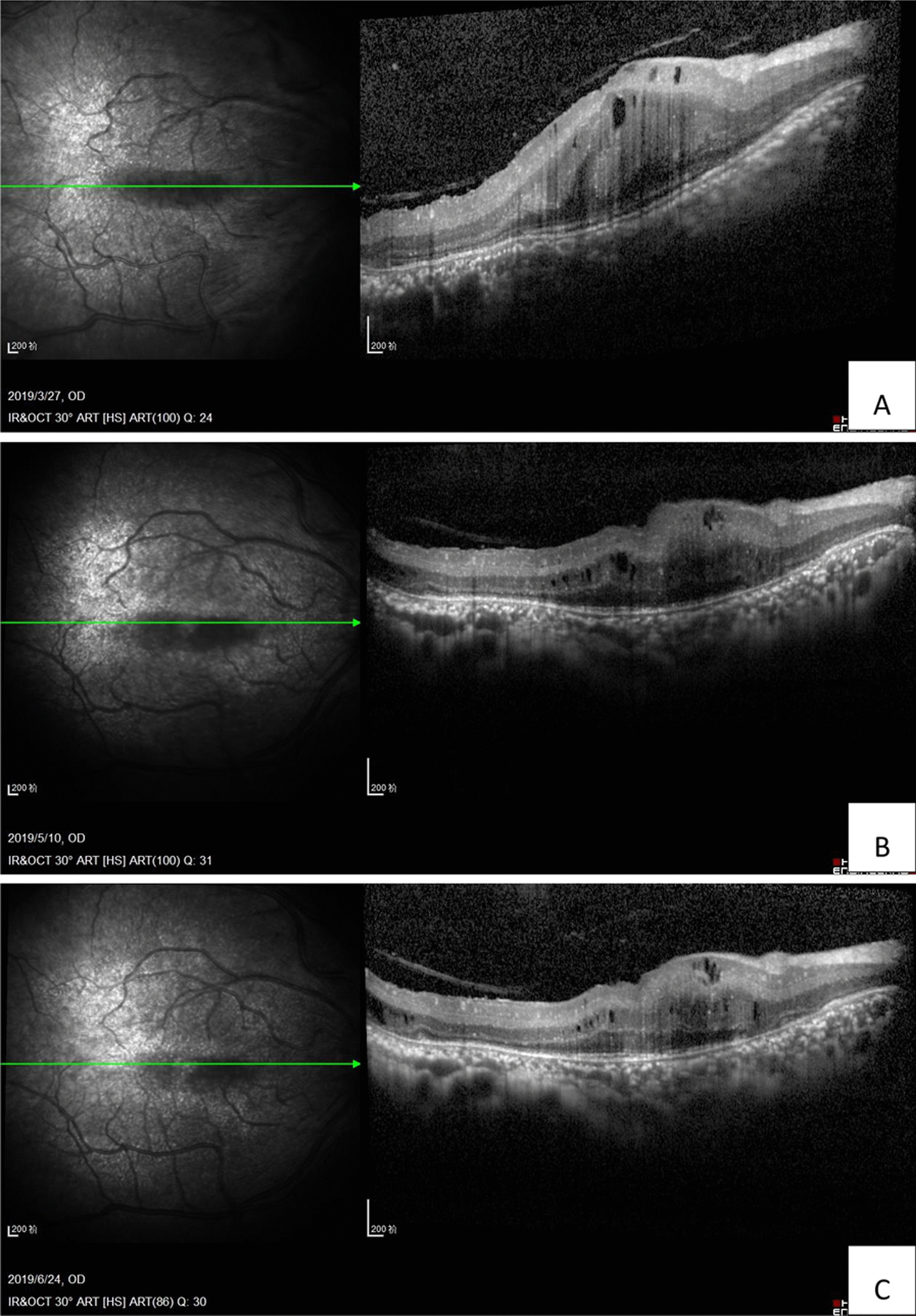
The OCT examination of right eye pretreatment (**A**), 1 month posttreatment (**B**) and 2 months posttreatment (**C**). The thickness of the choroid and retina reduced with time. The arrow in the left part OCT is just a indication on the fundus en face, that indicates exactly from which position the left part of OCT scan was made

**Fig. 5:**
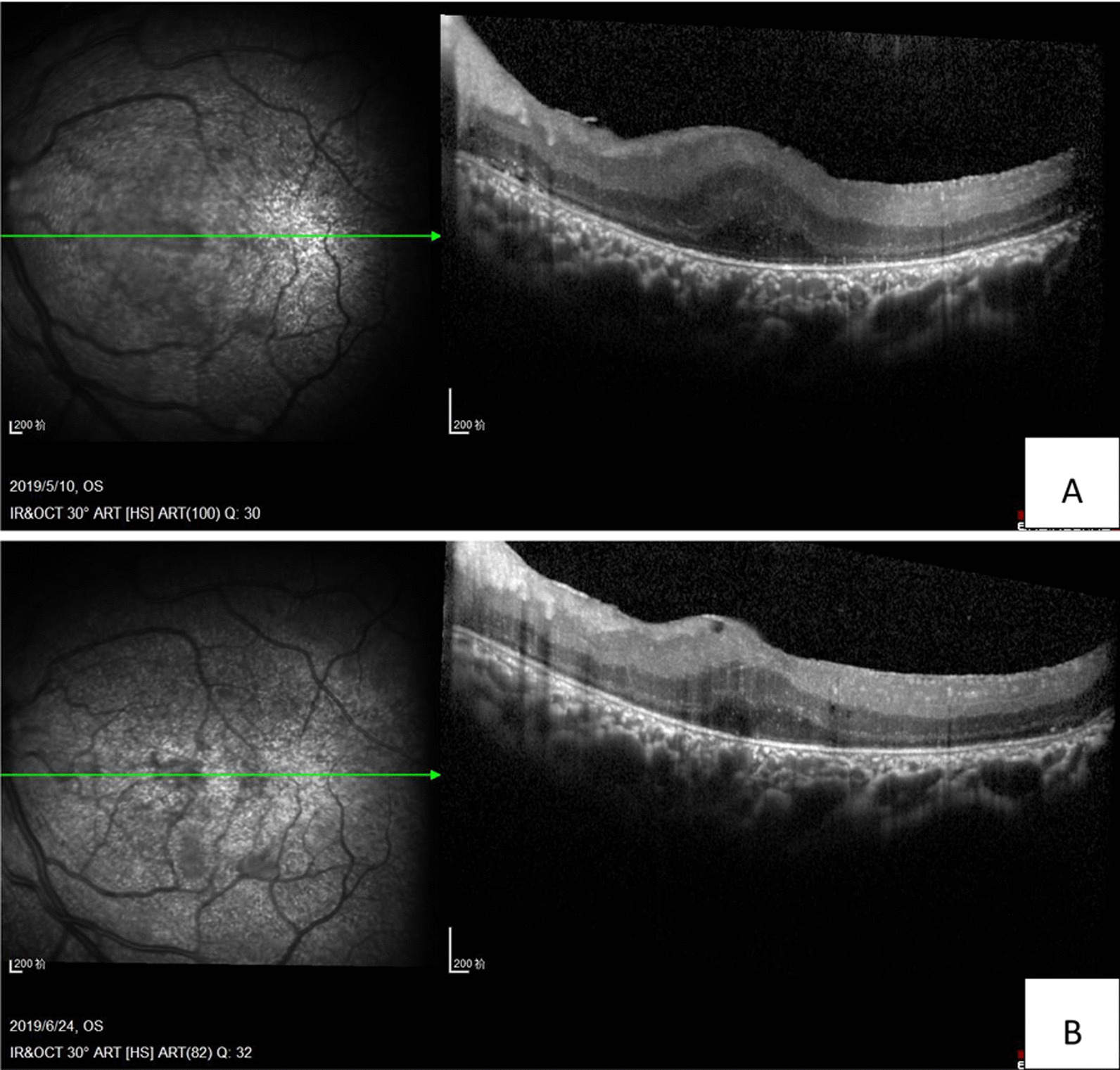
The OCT examination of left eye. It was impossible to conduct OCT examination pretreatment because of the total exudative retinal detachment, which located behind the lens. One month after first injection of ranibizumab (**A**) and 2 months posttreatment. **B** The thickness of retina and subretinal fluid reduced with time. The arrow in the left part OCT is just a indication on the fundus en face, that indicates exactly from which position the left part of OCT scan was made

Three days after the injection, the patient reported that his quality of vision had improved, although it was a minimal improvement that was not reflected on the Snellen visual chart. Careful examination revealed minor improvements in the extent of the retinal and choroidal detachments. Half a month after anti-VEGF treatment, the resolution of effusion in both eyes was significant. One month later, most of the effusion under the choroid had resolved, and the retinal detachment was further reduced (Figs. 2c, 3c).The VA of the right eye had improved from finger count to 0.05, which the patient reported was similar to his normal vision. The VA in the left eye improved up to approximately 0.03. The patient was very satisfied due to the remarkable improvement in his vision, and he became more confident in his daily life. Encouraged by this substantial improvement, and due to the slowing of the improvement process, the patient was willing to undergo a second injection 1 month after the first injection. Choroidal detachment was hardly observable 30 days after the second injection, and the subretinal effusion was completely absorbed on fundus examination with indirect ophthalmoscopy, +90 D preset lens with slit lamp and B-scan ultrasound. Optic coherence tomography (OCT) examinations also showed a reduction in retinal thickness and subretinal fluid (Figs. 4, 5). However, there was still a slight increase in the thickness of the choroid and sclera shown on the B-scan image, which appears largely attributable to the anatomic abnormality of nanophthalmos since it had remained stable 3 months after the second injection (Figs. 2e, 3e). The patient was born with very poor vision; although his VA was only approximately 0.05, he could live independently. Thus, sensitive treatment was required as any minor change in VA and subjectively visual quality would be critical. In the third month, the patient requested and received a third injection as he was anxious to avoid relapse. The reattachment of the choroid and retina remains to date approximately 2 years since the first intravitreal injection of ranibizumab. The other fundus appearances remain unchanged.

## Discussion and conclusions

UES can be classified into three types [5]. Type 1 involves nanophthalmic eyes in which the eyeball is small with an average axial length of 16 mm and is highly hypermetropic. Type 2 involves non-nanophthalmic eyes with clinically abnormal sclera and an average bulbar axial length more than 21 mm. Type 3 involves non-nanophthalmic eyes with clinically normal sclera. In our case, it was clear that the patient had type 1 UES. The axial length was 15.88 mm in the right eye and 16.21 mm in the left eye. Nanophthalmos is bilateral and may be sporadic or familial with either dominant or recessive patterns of inheritance [20]. Currently, the most popular explanation for the pathogenesis of UES associated with nanophthalmos is the abnormally thickened sclera and disordered collagen fibers that may lead to compression of the vortex vein and thereby lead to congestion of the choroidal vein and uveal effusion. Gass and Jallow [1] and Ward *et al.* [21] further suggested decreased scleral protein permeability as a cause of UES in these patients. They suggested that the disordered collagen fibers in the sclera of NO patients slow down the outflow of macromolecular substances, including proteins and glycosaminoglycans [7]. Jackson *et al.* [6] confirmed that effusion in the suprachoroidal space is osmotic fluid retention, in which protein escape from the choriocapillaries is a key factor. A recent study also revealed an increase in IL-6, IL-8, and VEGF expression in the aqueous humor of UES patients, which indicates that inflammation may be involved in the pathogenesis of UES and that abnormal permeability of choroid capillaries may also play an important role [8–10]. Although the exact or complete mechanism of UES remains to be clarified, congestion of the choriocapillaris and osmotic fluid retention-associated effusion are widely accepted theories; thus, surgical intervention is the most frequent method for treating UES. UES management includes partial or full/partial sclerectomy and surgical decompression of the vortex veins [11–13]. The surgical procedures have also been modified by using a fiberoptic-guided CO_2_ laser [22] to reduce bleeding and scarring, and partial sclerectomy has been combined with mitomycin C for improved success [23]. Uyama *et al.* [5] succeeded in treating six patients with partial sclerectomy combined with punch sclerotomy. Full-thickness sclerotomy without vortex vein decompression or sclerectomy has also been reported with good results [24].

In addition to the mainstream surgical procedures, medical treatment with various drugs has also been reported. Most of the medical treatments for UES have been described in case reports with limited patient numbers, and the underlying mechanisms remain unclear or have been described only on a hypothetical basis. The treatment results are not always consistent and have been shown to be contradictory in some cases. For example, it has been reported that systemic or topical steroids are not effective for UES in some studies, while others have shown very promising results. Shields *et al.* [15] reported the treatment of 104 eyes of patients with UES and found that 95% of patients could be managed with steroids, and only 5% needed scleral window surgery. Tong *et al.* [16] and Kumar *et al.* [25] also demonstrated that nonsteroidal antiinflammatory drugs (NSAIDs) can be effective for UES treatment. Park and Lee [17] reported a 64-year-old UES patient successfully treated with topical latanoprost and oral acetazolamide as well as topical bromfenac without surgery. Weinreb [18] demonstrated that topical prostaglandin administration could reduce sclera collagen levels by increasing scleral metalloproteinase levels. Derk *et al.* [26] reported two similar patients treated with oral carbonic anhydrase inhibitors and topical prostaglandin analogs. It has been suggested that carbonic anhydrase inhibitors may decrease macular edema by stimulating the pump mechanism of the retinal pigment epithelium. Furthermore, Guo *et al*. [10] reported the use of an anti-VEGF agent for the treatment of intractable UES in patients who had received a partial sclerectomy, but effusion was poorly absorbed or relapsed. They measured the concentration of IL-1β, IL-6, IL-8, IL-10, IL-12p70, tumor necrosis factor (TNF), and VEGF in the aqueous humor of three UES patients and found that IL-6, IL-8, and VEGF were elevated in all three cases. Of the three patients, one received three injections of ranibizumab with an interval of 4 weeks and another two received two injections of bevacizumab. Total absorption of the suprachoroidal and subretinal fluid was achieved in all three intractable UES patients at 2 weeks, 5 weeks, and 4 months. The exact underlying mechanism remains unclear. The researchers hypothesized that anti-VEGF agents can reduce the activation of macrophages and further regulate the expression of interleukin-6 (IL-6) and IL-8 [27, 28].

In our case, the decision to administer anti-VEGF agents was influenced by the report of Guo *et al.* [10], though it is difficult to find solid evidence to support the use of anti-VEGF agents at this time. The authors referred to the treatment as “empirical” use after the first effective treatment; however, the results were impressive. In this case, we faced a situation in which we had to act with uncertainty regarding the result. Neither the patient nor the doctor was prepared to risk further damage to the patient’s already poor vision. Therefore, trying ranibizumab was a reasonable and easy decision. Fortunately, positive results were achieved despite the unknown mechanisms. We believe that this result was very unlikely to be a spontaneous absorption of the effusion. The reason for this is that both eyes with severe UES rapidly responded to the anti-VEGF therapy and the effusion did not relapse in the 2-year follow-up period. Studies have demonstrated that VEGF can counteract inflammation and reduce choroidal hyperpermeability [27, 28]. The pathogenesis of UES is not fully understood. In many cases, the success of treatment was determined by theories based on assumptions. In addition, the answers to many interesting clinical questions related to UES remain unclear; for example, in type III UES [5], the eyeball axial length and sclera are all normal; thus, how does UES occur? In that case, the pathology does not match the theory of vortex vein compression or osmotic fluid retention. Second, according to the current understanding of congenital NO, the abnormal sclera thickness or disorganization of the collagen fibers is congenital; however, most UES arises in adult or middle age [3] and not at a very young age. It is also obvious that not every NO patient will experience UES; therefore, there must be other factors or triggers that lead to the occurrence of UES. There are reports that glaucoma, iridocyclitis, and other eye diseases could trigger UES [29], but our patient had none of these conditions. Furthermore, the function of VEGF or anti-VEGF is not fully understood. Studies have indicated that the permeability of microvessels is related to various inflammatory cytokines, such as IL-6, IL-8, chemokine monocyte chemoattractant protein 1 (MCP-1), and intercellular adhesion molecule-1 (ICAM-1)[30]. Inflammation could be an important cause for some idiopathic UES [31]. Kumar *et al.* [25] applied the indocyanine green chorioangiography (ICG) findings in two cases of UES to display focal areas of late-phase choroidal hyperfluorescence, which is suggestive of choroidal hyperpermeability. They treated these patients with NSAIDs and scattered lasers. Dvorak *et al.* [30] confirmed that VEGF can enhance microvascular hyperpermeability, which could also be a factor that causes macular edema in various retinal vascular diseases, such as branch vein occlusion. It is known that anti-VEGF can reduce the hyperpermeability of choroidal vessels [31, 32]. In the course of UES, if the hyperpermeability of choroidal vessels can be reduced, the suprachoroidal fluid may also decrease. All these studies mentioned above indicate that UES could be a multifactorial disease that involves congenital abnormal sclera, inflammation, and choroidal hyperpermeability. VEGF is likely to be involved in some of the pathogenesis of UES, and even though the mechanism is not exactly known, anti-VEGF agents can be applied. We believe it is important to be open to novel strategies and explore unknown treatments; however, there are insufficient studies to make definitive conclusions at present. We still report our experience with this patient, in whom we did not make any new egress for retained suprachoroidal fluid or apply drugs that were previously reported other than ranibizumab. At this moment, we can only suspect that the anti-VEGF agent may act by decreasing inflammatory elements, reducing the permeability of choroidal capillaries and other as-yet unknown mechanisms. Of course, this is only one patient case report. We hope to draw the attention of our colleagues to the use of anti-VEGF for UES and conduct future research.

We also determine a factor that could have acted as a trigger for the occurrence of UES in both eyes simultaneously. After a careful search and an analysis of any possible causes, we believe that the onset of UES in this patient may be related to a period of continuous extra physical stress preceding the abrupt vision deterioration.

It is worth further studying anti-VEGF agent treatment for UES patients as UES remains a major challenge for clinicians and intravitreal injection of anti-VEGF is a relatively safe and easy procedure, although we still do not know what percentage of UES patients can profit with this approach or how well patients will respond.

## Conclusion

Using anti-VEGF alone may be an effective and safe method for managing some types of UES, such as those resulting from nanophthalmos. Anti-VEGF can be applied before taking other more aggressive approaches, such as sclera window surgeries, which require profound surgical expertise and may cause more surgery-related complications. Further in-depth studies and clinical observations are needed to understand the use of anti-VEGF injections for UES and elucidate the underlying mechanism.

## Data Availability

All data generated or analyzed during this study are included in this manuscript.

## Abbreviations

UES: Uveal effusion syndrome
VEGF: Vascular endothelial growth factor
IL-6: Interleukin-6
IL-8: Interleukin-8
OD: Right eye
OS: Left eye
OU: Both eyes
C/D: Cup/disc
A/V: Artery/vein
IOP: Intraocular pressure
VA: Visual acuity
BCVA: Best corrected visual acuity
Kp: Keratic precipitates
FC: Finger count
NSAIDs: Nonsteroidal antiinflammatory drugs
OCT: Optic coherence tomography

## References

[1] Gass JD, Jallow S. Idiopathic serous detachment of the choroid, ciliary body, and retina (uveal effusion syndrome). Ophthalmology. 1982;89:1018–32. 10.1016/s0161-6420(82)34685-0 7177567

[2] Brockhurst RJ. Nanophthalmos with uveal effusion. A new clinical entity. Arch Ophthalmol. 1975;93:1989–99. 10.1001/archopht.1975.01010020923001 1200903

[3] Yang N, Jin S, Ma L, Liu J, Shan C, Zhao J. The pathogenesis and treatment of complications in nanophthalmos. J Ophthalmol. 2020;2020:6578750. 10.1155/2020/6578750 PMC7387986 32765903

[4] Elagouz M, Stanescu-Segall D, Jackson TL. Uveal effusion syndrome. Surv Ophthalmol. 2010;55:134–45. 10.1016/j.survophthal.2009.05.003 20159229

[5] Uyama M, Takahashi K, Kozaki J, Tagami N, Takada Y, Ohkuma H, *et al.* Uveal effusion syndrome: clinical features, surgical treatment, histologic examination of the sclera, and pathophysiology. Ophthalmology. 2000;107:441–9. 10.1016/s0161-6420(99)00141-4 10711879

[6] Jackson TL, Hussain A, Salisbury J, Sherwood R, Sullivan PM, Marshall J. Transscleral albumin diffusion and suprachoroidal albumin concentration in uveal effusion syndrome. Retina. 2012;32:177–82. 10.1097/IAE.0b013e318218a95a 21811210

[7] Gass JD. Uveal effusion syndrome. A new hypothesis concerning pathogenesis and technique of surgical treatment. Retina. 1983;3:159–63. 6635350

[8] Melincovici CS, Boşca AB, Şuşman S, Mărginean M, Mihu C, Istrate M, *et al.* Vascular endothelial growth factor (VEGF)—key factor in normal and pathological angiogenesis. Rom J Morphol Embryol. 2018;59:455–67. 30173249

[9] Takahashi H, Shibuya M. The vascular endothelial growth factor (VEGF)/VEGF receptor system and its role under physiological and pathological conditions. Clin Sci (Lond). 2005;109:227–41. 10.1042/CS20040370 16104843

[10] Guo J, Cao X, Li X. Partial thickness sclerectomy and intravitreal anti-VEGF therapy for intractable uveal effusion syndrome. Int Ophthalmol. 2019;39:1885–90. 10.1007/s10792-018-0992-1 30043134

[11] Mansour A, Stewart MW, Shields CL, Hamam R, Abdul Fattah M, Sheheitli H, *et al.* Extensive circumferential partial-thickness sclerectomy in eyes with extreme nanophthalmos and spontaneous uveal effusion. Br J Ophthalmol. 2019;103:1862–7. 10.1136/bjophthalmol-2018-313702 30877130

[12] Ozgonul C, Dedania VS, Cohen SR, Besirli CG. Scleral surgery for uveal effusion. Retina. 2017;37:1977–83. 10.1097/IAE.0000000000001521 28141752

[13] Wang BZ, Clark B, McKelvie P, Matthews BJ, Buttery RG, Chandra A. Four quadrant sclerotomies for uveal effusion syndrome. Eye (Lond). 2015;29:588–9. 10.1038/eye.2014.291 PMC4816367 25572577

[14] Johnson MW, Gass JD. Surgical management of the idiopathic uveal effusion syndrome. Ophthalmology. 1990;97:778–85. 10.1016/s0161-6420(90)32511-3 2374682

[15] Shields CL, Roelofs K, Di Nicola M, Sioufi K, Mashayekhi A, Shields JA. Uveal effusion syndrome in 104 eyes: response to corticosteroids—the 2017 Axel C. Hansen lecture. Indian J Ophthalmol. 2017;65:1093–104. 10.4103/ijo.IJO_752_17 PMC5700574 29133632

[16] Tong B, Wang C, Qi X. Unusual rapid resolution of postsclerectomy exudative retinal detachment with topical NSAIDs therapy in a case of nanophthalmos. J Int Med Res. 2020;48(8):300060519847376. 10.1177/0300060519847376 PMC7512026 31081412

[17] Park JH, Lee EK. Medical therapy for bilateral uveal effusion syndrome in nanophthalmos. Can J Ophthalmol. 2017;52:e199–201. 10.1016/j.jcjo.2017.04.017 29217039

[18] Weinreb RN. Enhancement of scleral macromolecular permeability with prostaglandins. Trans Am Ophthalmol Soc. 2001;99:319–43. PMC1359020 11797317

[19] Chan W, Fang-tian D, Hua Z, You-xin C, Rong-ping D, Ke T. Diagnosis and treatment of uveal effusion syndrome: a case series and literature review. Chin Med Sci J. 2011;26(4):231–620. 10.1016/s1001-9294(12)60006-6 22218051

[20] Sundin OH, Dharmaraj S, Bhutto IA, *et al.* Developmental basis of nanophthalmos: MFRPIs required for both prenatal ocular growth and postnatal emmetropization. Ophthalmic Genet. 2008;29(1):1–9. 10.1080/13816810701651241 PMC2739627 18363166

[21] Ward RC, *et al.* Abnormal scleral findings in uveal effusion syndrome. Am J Ophthalmol. 1988;106(2):139–46. 10.1016/0002-9394(88)90825-2. 2969684

[22] Bausili MM, Raja H, Kotowski J, Nadal J, Salomao DR, Keenum D, *et al.* Use of fiberoptic-guided CO_2_ laser in the treatment of uveal effusion. Retin Cases Brief Rep. 2017;11:191–4. 10.1097/ICB.0000000000000357 27768636

[23] Sabrosa NA, Smith HB, MacLaren RE. Scleral punch method with topical mitomycin C for safe revision of failed deep sclerectomy in nanophthalmic uveal effusion syndrome. Graefes Arch Clin Exp Ophthalmol. 2009;247:999–1001. 10.1007/s00417-008-1020-3 19130073

[24] Kong M, Kim JH, Kim SJ, Kang SW. Full-thickness sclerotomy for uveal effusion syndrome. Korean J Ophthalmol. 2013;27:294–8. 10.3341/kjo.2013.27.4.294 PMC3730074 23908578

[25] Kumar A, Kedar S, Singh RP. The indocyanine green findings in idiopathic uveal effusion syndrome. Indian J Ophthalmol. 2002;50:217–9. 12355699

[26] Derk BA, Benčić G, Corluka V, Geber MZ, Vatavuk Z. Medical therapy for uveal effusion syndrome. Eye (Lond). 2014;28:1028–31. 10.1038/eye.2014.126 PMC4135252 24903345

[27] Nakao S, Arima M, Ishikawa K, Kohno R, Kawahara S, Miyazaki M, *et al.* Intravitreal anti-VEGF therapy blocks inflammatory cell infiltration and re-entry into the circulation in retinal angiogenesis. Invest Ophthalmol Vis Sci. 2012;53:4323–8. 10.1167/iovs.11-9119 22661475

[28] Mirabelli P, Peebo BB, Xeroudaki M, Koulikovska M, Lagali N. Early effects of dexamethasone and anti-VEGF therapy in an inflammatory corneal neovascularization model. Exp Eye Res. 2014;125:118–27. 10.1016/j.exer.2014.06.006 24933712

[29] Burgoyne C, Tello C, Katz LJ. Nanophthalmia and chronic angle-closure glaucoma. J Glaucoma. 2002;11:525–8. 10.1097/00061198-200212000-00013 12483099

[30] Dvorak HF, Brown LF, Detmar M, Dvorak AM. Vascular permeability factor/vascular endothelial growth factor, microvascular hyperpermeability, and angiogenesis. Am J Pathol. 1995;146:1029–39. PMC1869291 7538264

[31] Diep MQ, Madigan MC. Choroidal detachments: what do optometrists need to know? Clin Exp Optom. 2019;102:116–25. 10.1111/cxo.12807 29971817

[32] Karimi S, Nourinia R, Mashayekhi A. Circumscribed choroidal hemangioma. J Ophthalmic Vis Res. 2015;10:320–8. 10.4103/2008-322X.170353 PMC4687268 26730320

